# Enhancing identification and diagnosis of nightmare disorder in active duty patients at a sleep disorders center: A quality improvement initiative

**DOI:** 10.1007/s44470-025-00016-0

**Published:** 2026-01-22

**Authors:** Amanda Radtke, Zahari Tchopev, Philip Cushman, James Kang, Aaron Burch, H. Samuel Scheuller, Matthew S. Brock

**Affiliations:** Department of Sleep Medicine, Wilford Hall Ambulatory Surgical Center, 1100 Wilford Hall Loop, 59th Medical Wing, JBSA-Lackland, Texas, 78236 USA

**Keywords:** Nightmares, Quality improvement, Screening, Diagnosis, Military, Military health, Parasomnia, Sleep disorder

## Abstract

**Study objectives:**

Nightmare Disorder (NDO) is an underdiagnosed sleep disorder causing significant impairment, particularly in active duty U.S. military personnel who experience unique stressors. Despite 59.28% of referred service members reporting nightmares and 19.76% screening positive for probable NDO, baseline diagnosis rate was only 0.60%. Our quality improvement project aimed to improve identification and diagnosis of NDO among active duty service members at our sleep disorders center by increasing the confirmed diagnosis rate from 0.60% to at least 5% within 12 months.

**Methods:**

A quality improvement initiative utilizing tiered Plan-Do-Study-Act cycles was implemented over seven months. Interventions included standardizing documentation of Nightmare Disorder Index results on polysomnography reports, targeted provider education, and chart flagging for patients screening positive for probable NDO. Diagnostic and process data were collected at baseline and following each intervention cycle.

**Results:**

The diagnosis rate of NDO increased from 0.60% at baseline to 9.12% after three Plan-Do-Study-Act cycles. The percentage of patients with probable NDO who were ultimately diagnosed (recognition rate) rose from 3.03% to 55.32%. Interventions were implemented without delaying care or increasing staff burden, surpassing the goal diagnosis rate of at least 5% within the target timeframe.

**Conclusions:**

Our interventions led to meaningful increases in NDO diagnoses that exceeded project goals. Given the significant effect of sleep disturbances and heightened mental health risks in the military, identification of this underdiagnosed disorder is clinically important and serves as the essential first step toward ensuring affected individuals receive appropriate treatment.

**Brief summary:**

**Current knowledge/study rationale**: Nightmare Disorder is significantly underdiagnosed in active duty service members despite causing substantial impairment and being highly prevalent in this population who face unique stressors. Sleep disorders centers often fail to identify and diagnose this condition even when service members report nightmares and screen positive on validated assessment tools.

**Study impact**: This quality improvement initiative demonstrates that systematic interventions including standardized screening documentation, provider education, and proactive chart flagging can dramatically increase nightmare disorder diagnosis rates in military healthcare settings. The successful implementation of these workflow-oriented processes provides a replicable model for other military and civilian sleep centers to improve identification of this underdiagnosed condition, serving as an essential first step toward ensuring affected patients receive appropriate treatment.

## Introduction

Sleep disorders are common in the active duty population and have been shown to dynamically reduce performance and quality of life [1–4]. Nightmare Disorder (NDO) is a Rapid Eye Movement (REM) sleep parasomnia characterized by extremely dysphoric, well-remembered dreams that usually involve threats to survival, security, or physical integrity and cause significant distress upon awakening. Diagnosis is confirmed when patients rapidly become oriented and alert upon awakening from the dysphoric dreams, and there is impairment in social, occupational, or other important areas of functioning because of the negative dream experience or nighttime disturbance of sleep [5].

Nightmares are categorized as being trauma-related or idiopathic, and they are conceptually and clinically distinct. Idiopathic nightmares occur without an identifiable traumatic trigger, and trauma-related nightmares replay specific events or contain similar themes to prior traumatic events, often being more severe and distressing than idiopathic nightmares [6–11]. The diagnostic criteria for NDO, as outlined in the International Classification of Sleep Disorders, Third Edition, Text Revision (ICSD-3-TR) and Diagnostic and Statistical Manual of Mental Disorders, Fifth Edition, Text Revision (DSM-5-TR), do not differentiate between these subtypes [5, 11].

Patients with nightmares have increased suicidality, worse self-reported and objective measures of sleep (shorter sleep duration, decreased sleep efficiency, longer sleep onset and REM latency), and higher rates of insomnia, anxiety, depression, and posttraumatic stress disorder (PTSD) [10, 12–19]. Despite their clinical relevance, nightmares are often overlooked in practice. Data indicate that clinicians frequently do not inquire about nightmares, and individuals affected by them rarely volunteer this information [4, 5]. Although NDO is estimated to impact at least 4% of the US adult population [20], the true prevalence is likely higher due to patterns of underreporting and underdiagnosis [4, 5].

Among active duty service members, nightmare prevalence is especially high. The military population is at increased risk for exposure to traumatic events, and they experience unique stress and trauma that can lead to chronic sleep disturbances, including trauma-related nightmares [6–10]. Importantly, military personnel have elevated rates of PTSD, insomnia, and obstructive sleep apnea (OSA), all of which can influence and complicate nightmare reporting and NDO diagnosis [1, 2, 21]. One study utilizing predeployment questionnaires found that 40% of active duty military experience current nightmares, without distinguishing between idiopathic and trauma-related nightmares [22]. In a clinical cohort of service members referred to our academic military sleep disorders center with sleep disturbances in 2016, 31.2% of those evaluated for sleep disturbances met criteria for NDO, trauma-related nightmares occurred in 60% of those patients, and only 3.9% reported nightmares as a reason for evaluation [23]. Despite its prevalence, NDO often goes undiagnosed and untreated in this population, with potential negative impacts on mental health, quality of life, and operational readiness. Disturbances in REM and Non-REM sleep, including nightmares, have been implicated in maladaptive responses to stress and trauma and may represent a modifiable risk factor for poor psychiatric outcomes [24]. Recognizing and addressing NDO is clinically important, as unaddressed sleep disturbances are associated with poorer outcomes, and sleep-focused interventions can improve psychiatric comorbidities such as PTSD, depression, and anxiety [24, 25].

One barrier to the diagnosis of NDO is that nightmares are a part of the diagnostic criteria for other conditions [11]. For example, intermittent nightmares are expected in Acute Stress Disorder and PTSD, and trauma-related nightmares are a hallmark symptom of PTSD [24, 26]. This may contribute to the underreporting and underdiagnosis of NDO in active duty patients. However, when posttraumatic nightmares require independent clinical attention, cause significant sleep disturbance, or persist even when other PTSD symptoms have remitted, then a diagnosis of NDO is appropriate per the ICSD-3-TR [5].

Given the well-documented associations between nightmares and adverse clinical outcomes, accurate identification and diagnosis of NDO in military personnel is essential. At our sleep disorders center, the baseline rate of NDO diagnosis among referred active duty service members was 0.60%, highlighting a substantial gap compared to estimated prevalence rates in both the general population and military cohorts. Currently, no quality measures report on the rate of confirmed diagnosis of NDO.

This quality improvement project sought to address the barriers to effective identification and diagnosis of NDO and improve the NDO diagnosis rate by utilizing tiered Plan-Do-Study-Act cycles. Advocated by organizations like the Joint Commission on Accreditation of Healthcare Organizations and the Institute for Healthcare Improvement (IHI), the Plan-Do-Study-Act cycle is an iterative, four-step problem-solving approach widely used in quality improvement initiatives that enables teams to test and refine changes in a structured manner, leading to continuous improvement in process and outcomes [27, 28]. While there are no specific examples of Plan-Do-Study-Act cycles being used to improve NDO diagnosis in sleep medicine to date to the authors’ knowledge, the approach has been successfully applied to improve countless different outcomes and processes in medicine and other industries. Our SMART (specific, measurable, achievable, relevant, and time-bound) goal was to increase the diagnosis rate of NDO from 0.60% to at least 5% among active duty service members referred to our sleep disorders center within 12 months. This goal considered the estimated prevalence of NDO in the general U.S. adult population and the increased prevalence in active duty military.

## Methods

This quality improvement project was conducted at our academic military sleep disorders center, which provides specialized care for military personnel with sleep complaints. Referrals to our sleep disorders center are made as part of standard clinical pathways from other clinics, predominantly primary care clinics. None of the study authors were involved in the referral process. As part of the standard evaluation process after referral, active duty service members participate in a group intake appointment, during which they complete a series of questionnaires. Their responses help determine the need for further diagnostic testing, such as polysomnography (PSG), prior to their in-office consultation.

Among the intake tools is the Nightmare Disorder Index (NDI), a validated 5-item self-report questionnaire designed to screen for NDO in accordance with the DSM-5 diagnostic criteria [29]. The first question quantifies how many nights per week they experienced nightmares in the past month, from 0 (“0 nights per week”) to 4 (“7 nights per week”). Questions 2–4 utilize a 4-point Likert scale from 0 (“never” or “not at all”) to 4 (“always” or “very much”) and measure how often nightmares cause distress, impairment, and waking and quickly becoming alert. A score of ≥ 1 on question 1 and ≥ 2 on questions 2–4 suggests probable NDO. The final question quantifies the duration the nightmares have been bothersome, from 0 (“ < 1 week”) to 4 (“ > 12 months”), serving as an acuity specifier. It is important to note that the NDI is meant to be a screening tool, and a diagnosis of NDO cannot be made without a clinical interview. Participants could only be diagnosed with NDO after a clinic appointment with a diagnosing clinician at our sleep disorders center. While some study authors are also physicians in the clinic, the diagnostic criteria from the ICSD-3-TR for NDO were used to make the diagnosis based on information obtained during the clinical interview.

For the purposes of this study, our definition of NDO includes both idiopathic and trauma-related nightmares. This decision was based on several factors. First, the diagnostic criteria for NDO in the ICSD-3-TR and DSM-5-TR do not differentiate between these subtypes [5, 11]. Second, in a clinical setting, especially within a military population, it can be challenging to definitively determine the etiology of nightmares. Many military personnel have experienced traumatic events and other stressors that could contribute to nightmares, and some service members do not want to divulge the nature of their nightmares [30]. Finally, our primary aim was to improve the overall identification and treatment of NDO, regardless of the specific cause.

For baseline data, encounters of referred patients who attended initial intake appointments in May 2024 were reviewed. The primary outcome measure was the percentage of referred active duty service members diagnosed with NDO at their consultation appointment in clinic (diagnosis rate = number of active duty service members diagnosed with NDO/number of active duty service members who went through the group intake appointment). Additional data collected includes active duty status, number of patients going through each initial group appointment, number/percentage of service members who completed the NDI, number/percentage of service members with probable NDO based on NDI results, number/percentage of service members who reported nightmares in the past month based on NDI results, number/percentage of service members who reported nightmares as a chief complaint, and the number of patients diagnosed with NDO at their in-office consultation in the sleep disorders center. A secondary outcome measure was the number of service members with probable NDO who were diagnosed with NDO (recognition rate = number of active duty service members diagnosed with NDO/number of active duty service members with probable NDO based on the NDI).

**Fig. 1:**
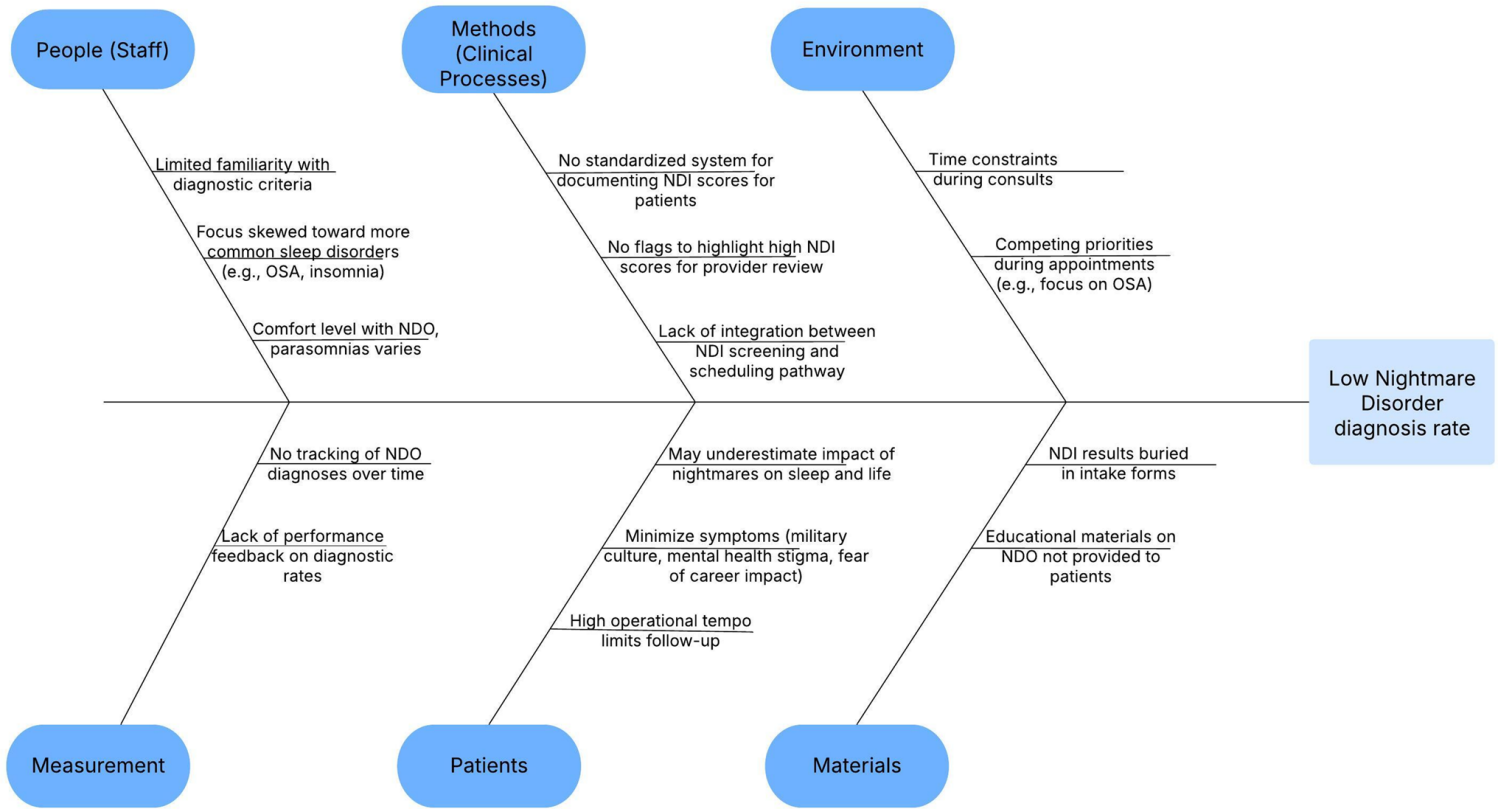
Ishikawa/fishbone cause and effect diagram. This diagram identifies and categorizes the causes contributing to the low NDO diagnosis rate at our Sleep Disorders Center. By identifying root causes, targeted interventions could be developed to increase the recognition and diagnosis of NDO. *NDI *Nightmare Disorder Index, *NDO *nightmare disorder, *OSA *obstructive sleep apnea

**Fig. 2:**
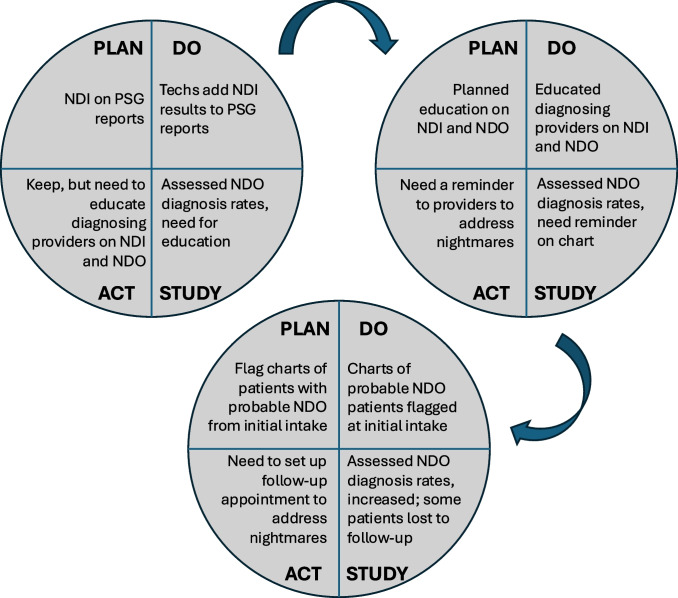
PDSA cycles to enhance identification and diagnosis of NDO in active duty service members. *PDSA* Plan-Do-Study-Act, *NDI* Nightmare Disorder Index, *NDO* nightmare disorder, *PSG* polysomnogram

Following baseline data acquisition, this project involved a multidisciplinary team at the sleep disorders center, including diagnosing providers (faculty and fellow physicians, physician assistants), sleep technicians, sleep lab coordinators, and nurses. Contributing causes to the low NDO diagnosis rate were identified and categorized (Fig. 1), and utilizing the Model for Improvement framework, we conducted iterative Plan-Do-Study-Act cycles [27]. Three key interventions were implemented with the goal of improving the identification and diagnosis of NDO (Fig. 2).

First, we had sleep technicians add NDI results to the top of PSG reports, standardizing documentation of these results, which previously did not have a place where they were routinely documented in a patient’s chart. This standardized documentation of the NDI results alongside existing screening data included on PSGs, including scores of the Insomnia Severity Index and Epworth Sleepiness Scale. Upon review of data and discussion with the team after implementing this intervention, we identified that diagnosing providers would benefit from education. As a second intervention, targeted education was delivered to the diagnosing providers of the clinic. This was a 60 min training during a staff meeting. The topics included the proper diagnosis of and coding for NDO (especially regarding diagnosis in the setting of comorbid psychiatric disorders, such as PTSD) and the interpretation of NDI results to identify patients with probable NDO. After evaluation of our solution and planning, we implemented our third intervention: physically marking the paper charts of patients with probable NDO so that diagnosing providers were prompted to evaluate for nightmares in the consultative clinic appointment. Timing of interventions was as follows: Plan-Do-Study-Act cycle 1 July 22, 2024, Plan-Do-Study-Act cycle 2 September 10, 2024, and Plan-Do-Study-Act cycle 3 December 2, 2024. Data collection occurred weekly after each Plan-Do-Study-Act cycle for 6–7 weeks. To explore the relationship between time after each intervention and NDO diagnosis rates, Spearman correlation coefficients were calculated.

## Results

At baseline (May 2024), 167 active duty service members (100%) completed the NDI, 59.28% reported nightmares within the past month (answered ≥ 1 on the first question of the NDI), 19.76% had probable NDO, 0.60% reported nightmares as a chief complaint, the recognition rate was 3.03%, and the diagnosis rate was 0.60%. After the first intervention, 306 service members (100%) completed the NDI, 77.78% reported nightmares within the past month, 22.55% had probable NDO, 2.94% reported nightmares as a chief complaint, the recognition rate was 13.04%, and the diagnosis rate was 2.94%. Following the second intervention, 509 service members (100%) completed the NDI, 72.10% reported nightmares within the past month, 24.17% had probable NDO, 3.93% reported nightmares as a chief complaint, the recognition rate was 5.69%, and the diagnosis rate was 1.38%. After the third intervention, 285 service members (100%) completed the NDI, 53.68% reported nightmares within the past month, 16.49% had probable NDO, 7.37% reported nightmares as a chief complaint, the recognition rate was 55.32%, and the diagnosis rate was 9.12% (Table 1). The Spearman (r) correlation coefficients for each intervention were as follows: −0.64 for Plan-Do-Study-Act cycle 1, −0.87 for Plan-Do-Study-Act cycle 2, and 0.14 for Plan-Do-Study-Act cycle 3.

**Table 1:** Results of screening for nightmares and probable NDO, clinical diagnosis rate of NDO, and recognition rate of NDO

	ADSMs screened with NDI	ADSMs with nightmares (%)	ADSMs with probable NDO (%)	ADSMs diagnosed with NDO (%)	Recognition rate (%Probable NDO diagnosed with NDO)
Baseline	167	99 (59.28)	33 (19.76)	1 (0.60)	3.03%
PDSA 1	306	238 (77.78)	69 (22.55)	9 (2.94)	13.04%
PDSA 2	509	367 (72.10)	123 (24.17)	7 (1.38)	5.69%
PDSA 3	285	153 (53.68)	47 (16.49)	26 (9.12)	55.32%

These are data for tracked measures at baseline and after each PDSA cycle

*ADSMs* active duty service members, *PDSA* Plan-Do-Study-Act, *NDI* Nightmare Disorder Index, *NDO* nightmare disorder

**Fig. 3:**
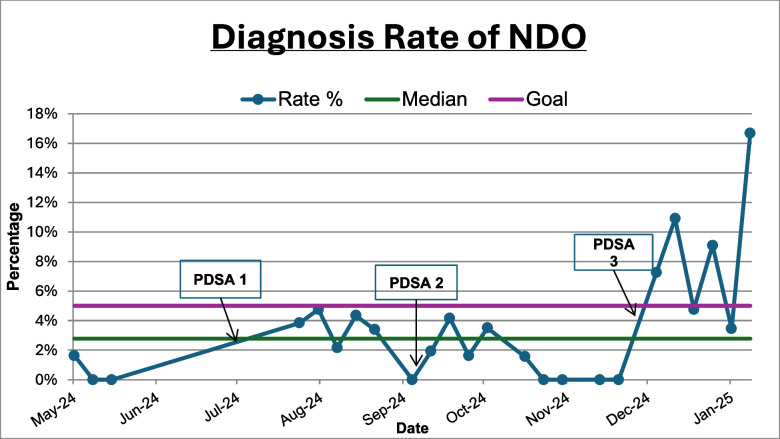
Diagnosis rate of NDO run chart. This displays the percentage of active duty service members (ADSMs) diagnosed with nightmare disorder (NDO) (Diagnosis Rate) at our SDC from May 2024 to January 2025. The desired direction of data is up. The rate (%) is shown by the dark blue line with circular markers, indicating the percentage of ADSMs diagnosed with NDO. The median (2.78%) is represented by the gray line. The goal line in light blue is set at 5%, reflecting the project’s target for diagnosis rates. The final data point reached 16.67%, the highest observed recognition rate during the measurement period. *ADSMs* active duty service members, *NDO* nightmare disorder, *SDC* Sleep Disorders Center, *PDSA* Plan-Do-Study-Act

**Fig. 4:**
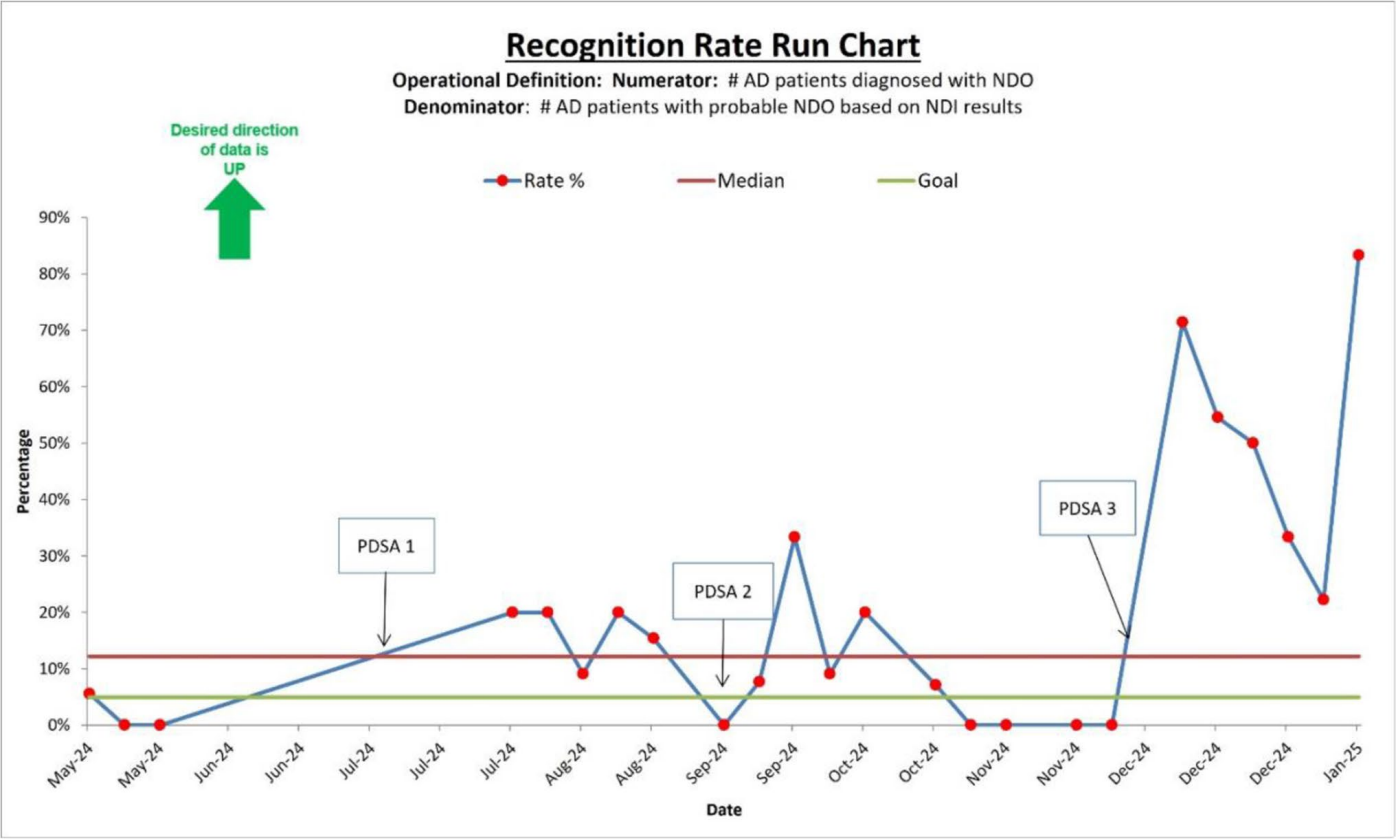
Recognition rate run chart. This displays the Recognition rate (percentage of ADSMs with probable NDO who were diagnosed with NDO at their in-clinic appointment) at our SDC from May 2024 to January 2025. The desired direction of data is up. The rate % is shown by the dark blue line with red circular markers. The median (12.2%) is represented by the red line. The goal line in green is set at 5%. The final data point in January 2025 reached 83.33%, the highest observed recognition rate during the measurement period. *AD* active duty, *ADSMs* active duty service members, *NDO* nightmare disorder, *PDSA* Plan-Do-Study-Act, *SDC* Sleep Disorders Center

**Fig. 5:**
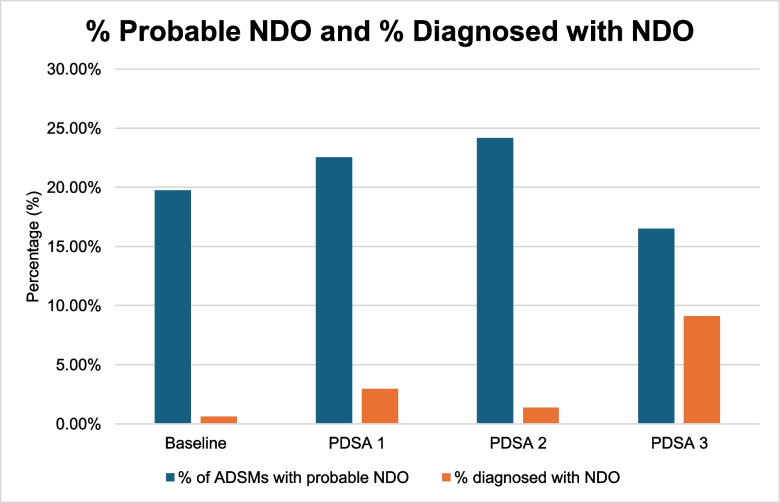
Percentage of ADSMs with probable NDO compared to the percentage of ADSMs diagnosed with NDO. The difference between the percentage of ADSMs with probable NDO compared to the percentage of ADSMs diagnosed with NDO was smallest after PDSA cycle 3 (7.37%) compared to baseline (19.16%), PDSA 1 (19.61%), and PDSA 2 (22.79%). *ADSMs* active duty service members, *NDO* nightmare disorder, *PDSA* Plan-Do-Study-Act

Overall, the diagnosis rate (percentage of active duty service members who were diagnosed with NDO at their clinical consultation appointment) increased from 0.60% at baseline to 9.12% after the third intervention (Fig. 3). The recognition rate (active duty service members diagnosed with NDO at their clinical consultation appointment/active duty service members with probable NDO based on NDI results) increased from a baseline of 3.03% to 55.32% after the third Plan-Do-Study-Act cycle (Fig. 4). The above interventions were implemented without adding excessive delays or backlogs in clinic appointments. A total of 12 diagnosing providers participated in targeted education on the NDI and NDO. Over the course of the project, we achieved our goal of improving NDO diagnosis rates to at least 5% within 12 months (Fig. 5).

## Discussion

This Quality Improvement project sought to improve NDO diagnosis rates in our sleep disorders center by addressing some of the barriers to identification and diagnosis of NDO. Initially, NDO diagnosis rates were low and inconsistent. Our first (July 2024) and second (September 2024) interventions led to minor, short-lived improvements in NDO diagnosis rates. Our first intervention, standardizing documentation of NDI results on PSG reports, was meant to cut down time of chart review and make results visible and actionable by diagnosing providers. There was a slight increase in diagnosis rates after the first intervention, which started below 1% in May 2024 and gradually climbed to around 3–4% in August, but the diagnosis rate returned to 0% in September 2024.

Following our second intervention, education of diagnosing providers, there was no sustained increase, with the highest diagnosis rate of 4.17% in September 2024 and rates dropping back to 0% by November 2024. A single educational session alone was not sufficient to improve NDO diagnosis rates. This could be due to multiple factors, including potential resistance to change, time constraints limiting translation of knowledge into practice, and the need for more comprehensive system-level changes. Prior studies have shown that training sessions spread out over time, or spaced education, leads to an improvement in long-term retention versus a single educational session [31–33]. One limitation of our project is that we did not conduct any assessments to ensure providers gained the goal knowledge from the educational session provided or to gauge willingness to change practice based on the session.

The third intervention, flagging charts of patients with probable NDO based on NDI results, appeared much more effective. A notable improvement occurred after our third intervention, suggesting that changes made during Plan-Do-Study-Act cycle 3 had a strong positive impact on diagnosing NDO in active duty service members. After the third intervention, there was a sharp increase in diagnosis rates, with 6 data points above the median and multiple data points exceeding the goal line. The final data point in January 2025 reached 16.67%, the highest observed rate during the measurement period. Plan-Do-Study-Act cycle 3 was the most impactful, increasing the percentage of active duty service members diagnosed with NDO above both the median and our project’s goal. The rise in diagnosis rates suggests that the third intervention may be effective long-term, but additional data collection is necessary to confirm sustained improvement and a shift above the median.

The negative Spearman correlation coefficients for Plan-Do-Study-Act cycles 1 and 2 align with the decreasing diagnosis rates over time following these interventions, with a more pronounced decline after provider education (Plan-Do-Study-Act cycle 2). In contrast, the Spearman correlation coefficient for Plan-Do-Study-Act cycle 3 being closer to 0 implies that the diagnosis of NDO was unaffected by time after this intervention. This suggests a more sustained impact from chart flagging, though other factors could have influenced diagnosis rates as well. These findings support the idea that workflow-oriented processes may promote more lasting changes in clinical practice than a single educational session.

Ultimately, we increased NDO diagnosis rates from 0.60% to 9.12% after 3 Plan-Do-Study-Act cycles within 7 months, exceeding the goal of at least 5% within 12 months. Our secondary outcome, NDO recognition rate, increased from 3.03% at baseline to 55.32% after the third Plan-Do-Study-Act cycle. The percentage of active duty service members with probable NDO based on NDI results ranged from 16.49% to 24.17%, suggesting NDO is quite prevalent in the service members referred to our sleep disorders center, and recognition rates increased after our interventions. It is possible that the third intervention ‘built upon’ the first two in that we flagged patients’ charts who had probable NDO, and the diagnosing providers now had a standardized place to find NDI results and had the knowledge, after our educational session, regarding how to interpret those NDI results and the diagnostic criteria and proper coding of NDO.

Our findings must be interpreted considering several limitations. First, increasing diagnostic awareness may have contributed to observer bias. Second, we did not track the prevalence, referral rates, or diagnosis rates of comorbidities such as OSA, PTSD, and insomnia across each PDSA cycle. This lack of comorbidity data limits our ability to fully assess the potential for misdiagnosis (e.g., NDO being misdiagnosed as insomnia or vice versa, or nightmares being a symptom of OSA) or to understand the influence of comorbidities on nightmare reporting and diagnosis rates. Third, while diagnosis rates increased following Plan-Do-Study-Act cycles 1 and 2, they declined modestly in subsequent months, raising questions about sustainability. Finally, as some authors are clinicians at the study site, there is potential for unconscious bias in diagnoses, though independent clinical interviews were required for each case.

The diagnostic boundary between NDO and other conditions, such as PTSD, is challenging in practice. The DSM-5 requires that coexisting mental disorders and mental conditions do not adequately explain the predominant complaint of dysphoric dreams [11]. According to these diagnostic criteria, clinicians must determine whether PTSD or another disorder fully accounts for nightmares, which is difficult to assess for multiple reasons. First, it would require a thorough diagnostic interview to evaluate for other conditions such as PTSD, which is not always feasible. Additionally, there is no research to guide when another condition “adequately explains” the dysphoric dreams. Third, the DSM-5 diagnostic criteria for PTSD include recurrent distressing dreams related to the traumatic event [11]. However, the inherently subjective nature of this criterion presents challenges in definitively identifying a dream as being of the traumatic event and whether this determination relies on the patient’s perspective or the clinician’s interpretation. These challenges are important issues that should be considered in future revisions of diagnostic nosology.

Even after three Plan-Do-Study-Act cycles, there remains a 7.37% gap between active duty service members with probable NDO and those diagnosed with NDO. We did not more closely review these cases to determine the cause of the discrepancy. Therefore, the current processes will remain in place, with future Plan-Do-Study-Act cycles planned to further enhance the identification and diagnosis of NDO in our active duty population. Future staff at our sleep center will need to be trained in the current processes, and education regarding NDO and the NDI will be incorporated into onboarding time of future incoming physicians.

Our approach is likely generalizable to all sleep centers to increase NDO diagnosis rates, although some limitations should be considered. First, we recognize that paper charts, while integral to our sleep disorders center’s workflow alongside the electronic health record (EHR), are not universally used. For practices without paper charts, we recommend using flags or alerts within the EHR as an alternative intervention. There will likely always be a discrepancy between those identified with probable NDO based off the screening NDI and those ultimately diagnosed with NDO. This is due to the fact that NDO remains a clinical diagnosis requiring an interview with the patient as questionnaire results alone are often unreliable, especially in the setting of fluctuating frequency and severity of nightmares [34]. Additionally, there is variability in how providers conduct clinical consultation appointments, and there might not be buy-in and practice change from all providers. Diagnosing providers may not ask about nightmares, as prior evidence has shown, or address the NDI results, even if they show probable NDO on screening.

The possible increases in encounter times and increase in staff workload are balancing measures that should be considered prior to implementing these interventions in another medical setting. Informal feedback and electronic medical record-based time tracking revealed no adverse workflow effects, and patient feedback reports showed no complaints after any of our interventions were implemented.

In the most recent stage of planning after the third Plan-Do-Study-Act cycle, the team has brainstormed several potential interventions. One future intervention will involve sleep center personnel scheduling patients with probable NDO for follow-up within 2–3 months from initial intake to decrease the number of patients lost to follow-up. An additional potential intervention includes creating patient-facing resources, including flyers in exam rooms and/or pamphlets in the waiting area, giving some facts about NDO, its impact on sleep and other aspects of life, and encouraging patients to discuss any nightmares in the appointment with their diagnosing provider. Another future intervention includes spaced education, including quarterly short review sessions on NDO and the NDI, incorporated into faculty meetings.

As NDO diagnosis rates improve, treatment implications are crucial. Although evidence-based therapies like Imagery Rehearsal Therapy (IRT) and Cognitive Behavioral Therapy for Nightmares (CBT-N) are available to active duty military personnel through our collaboration with clinical health psychology, provider availability can limit access to these beneficial services [20, 35, 36]. Pharmacological interventions, such as prazosin, are also an option, particularly in cases stemming from trauma, although their effectiveness varies [20, 37, 38]. To improve patient outcomes, future efforts should concentrate on streamlining treatment pathways and ensuring timely access to personalized care following diagnosis.

Through systematic Plan-Do-Study-Act cycles, our quality improvement project successfully increased the NDO diagnosis rate among active duty service members referred to our sleep disorders center from 0.60% to 9.12%, surpassing our goal of at least 5% within 12 months. Our sequential interventions, including standardized documentation of screening results, targeted provider education, and proactive chart-based flagging, collectively overcame barriers to identification and diagnosis of NDO. Nonetheless, limitations related to possible diagnostic bias, comorbidity overlap, and sustainability warrant careful consideration. Future initiatives should incorporate longitudinal comorbidity tracking, diagnostic reliability assessments, and structured follow-up to ensure durable improvements. Our most impactful intervention was the proactive chart flagging intervention, highlighting the importance of workflow-oriented clinical processes. This initiative underscores the necessity of systematic screening and diagnosis of NDO to improve sleep quality, mental health, and operational effectiveness in military personnel [39].

The ability to identify and diagnose NDO is needed prior to treatment, which can lead to improvements in sleep quality, mental health, quality of life, readiness, and mission effectiveness. Future projects can explore the next step, ensuring adequate treatment of NDO after identification and diagnosis. Due to the substantial impact of sleep disturbances and elevated mental health risks within the military population, recognizing this underdiagnosed disorder is a critical clinical priority and represents a foundational step in connecting affected individuals with effective treatment.

## Data Availability

Not applicable.

## Abbreviations

DHA: Defense Health Agency
DSM-5-TR: Diagnostic and Statistical Manual of Mental Disorders, Fifth Edition, Text Revision
EHR: Electronic health record
ICSD-3-TR: International Classification of Sleep Disorders, Third Edition, Text Revision
NDI: Nightmare Disorder Index
NDO: Nightmare disorder
OSA: Obstructive sleep apnea
PTSD: Posttraumatic stress disorder
REM: Rapid eye movement
